# The Effect of Intrapartum Antibiotic Prophylaxis at Caesarean Section on Childhood Obesity: A Quasi‐Experimental Study in Two Bi‐Ethnic UK Cohorts

**DOI:** 10.1111/ppe.70179

**Published:** 2026-08-04

**Authors:** Gillian Santorelli, Lucy Pembrey, Sergio Souza da Cunha, Emily S. Petherick, Ellena Badrick, Lila Goodwin, Sam Oddie, Neil Pearce, John Wright

**Affiliations:** ^1^ Bradford Institute for Health Research Bradford Teaching Hospitals NHS Foundation Trust Bradford UK; ^2^ Department of Medical Statistics London School of Hygiene & Tropical Medicine London UK; ^3^ School of Sport, Exercise and Health Sciences Loughborough University Loughborough UK; ^4^ National Institute for Health Research (NIHR) Leicester Biomedical Research Centre, University Hospitals of Leicester NHS Trust and University of Leicester Leicester UK

**Keywords:** antibiotic prophylaxis, caesarean section, childhood obesity, cohort study, ethnicity, quasi‐experimental study

## Abstract

**Background:**

Clinical guidelines now recommend administering intrapartum antibiotic prophylaxis (IAP) before skin incision at caesarean section to prevent maternal infection. However, this practice exposes the fetus to antibiotics, raising concerns about potential long‐term effects on the infant microbiome and the risk of childhood obesity.

**Objectives:**

To assess whether the timing of IAP at caesarean section—before skin incision versus after umbilical cord clamping—is associated with childhood obesity at age 4–5 years.

**Methods:**

We conducted a quasi‐experimental study of a hospital‐wide policy change in clinical practice using data from two birth cohorts (Born in Bradford [BiB] and Born in Bradford's Better Start [BiBBS]). The study included 1985 children of White British or Pakistani heritage born by caesarean section between 2007 and 2019. Children exposed to pre‐incision IAP (*n* = 324) were compared with those unexposed (post‐cord clamping IAP; *n* = 1661). The primary outcome was obesity (BMI z‐score > 95th percentile) at age 4–5 years. Adjusted Risk Ratios (aRR) were estimated using multivariable Poisson regression stratified by ethnicity.

**Results:**

The prevalence of obesity was 11.9%. Adjusted risk ratios for obesity were 1.25 (95% CI 0.53 to 2.98) for White British children and 1.25 (95% CI 0.64 to 2.43) for Pakistani children. Similarly, estimates for BMI z‐score had wide confidence intervals indicating, limited precision.

**Conclusions:**

We did not observe a clear difference in childhood obesity at 4–5 years between pre‐incision and post‐cord clamping prophylactic antibiotics at caesarean section. Confidence intervals were wide, and modest clinically relevant effects cannot be excluded. Findings are compatible with no large adverse effect and may provide reassurance regarding the metabolic safety of current clinical practice.

## 1 Introduction

In England, the rate of caesarean sections (CS) has increased substantially, from 25% in 2011–12 to 45% in 2024–25 [1]. A key component of maternal safety during CS is the administration of prophylactic antibiotics to prevent surgical site infection. Historically, antibiotics were administered after umbilical cord clamping. However, following a Cochrane review in 2010 and subsequent guidance from the National Institute for Health and Care Excellence (NICE), UK clinical practice changed in 2011 to recommend administration prior to skin incision, reducing maternal wound infection by 30%–40% [2, 3].

While beneficial for maternal outcomes, this change results in direct foetal exposure to antibiotics during a critical developmental window for the establishment of the infant gut microbiome [4]. Antibiotics administered prior to incision cross the placenta rapidly, resulting in measurable foetal serum concentrations before cord clamping [5, 6]. The early‐life microbiome plays a central role in immune development and metabolic programming [7], and disruption of this ecosystem has been hypothesised to contribute to long‐term health outcomes, including obesity and other non‐communicable diseases [8].

Findings from studies linking early‐life antibiotic exposure to childhood obesity are mixed. Meta‐analyses have reported associations with postnatal antibiotic exposure [9, 10], and more recent studies suggest that intrapartum antibiotic prophylaxis (IAP), particularly for Group B Streptococcus (GBS), may be associated with increased childhood BMI [11, 12, 13]. However, previous analyses in the Born in Bradford (BiB) cohort did not detect a clear association with maternal antibiotic exposure during pregnancy [14], and the specific impact of antibiotic timing at CS has not been well characterised. Overall, the certainty of evidence in this area remains low [10].

This study aimed to assess whether the timing of IAP at CS is associated with (1) obesity at 4–5 years, and (2) body mass index (BMI) z‐score at 4–5 years. We used data from the Born in Bradford research programme to conduct a quasi‐experimental comparison arising from a hospital‐wide policy change at Bradford Royal infirmary. Because variation in exposure was driven by policy rather than individual clinical decision‐making, this design reduces confounding by indication compared with conventional observational comparisons.

## 2 Methods

### 2.1 Study Design and Setting

We conducted a quasi‐experimental study arising from a hospital‐wide change in the timing of intrapartum antibiotic prophylaxis (IAP) at caesarean section (CS) at Bradford Royal Infirmary. Prior to 2011, prophylactic antibiotics were routinely administered after umbilical cord clamping. Following national guidance, clinical practice transitioned to administration before skin incision. This policy change created two exposure groups defined by calendar period rather than individual clinical characteristics.

### 2.2 Study Population

Participants were drawn from two Bradford‐based prospective birth cohorts: Born in Bradford (BiB) and Born in Bradford's Better Start (BiBBS). The BiB cohort recruited 12,453 pregnant women between 2007 and 2011 and includes births spanning the period during which the hospital policy on IAP timing transitioned from post‐cord clamping to pre‐incision administration [15, 16]. As such, this cohort includes both exposed and unexposed children. The BiBBS cohort recruited 5758 women between 2016 and 2024 from three inner‐city areas of Bradford characterised by high levels of socioeconomic deprivation [17]. Recruitment occurred after full implementation of the pre‐incision antibiotic policy. Consequently, children born by CS in this cohort were expected to have been exposed to pre‐incision IAP. This was supported by a review of a random sample of maternity records (*n* = 59) of maternal records. Although the Better Start programme includes a range of early‐years interventions, these were not implemented during the perinatal period for the children included in this analysis and therefore could not have influenced exposure status or early‐life outcomes relevant to this study.

### 2.3 Inclusion and Exclusion Criteria

For this analysis, we included liveborn singleton children delivered by caesarean section between 2007 and 2019 who were followed up to 4–5 years. BiBBS births were restricted to those occurring up to 2019 to allow complete outcome ascertainment. Where a mother contributed more than one eligible pregnancy, each child was included. For multiple births (e.g., twins), only the first‐born child was retained. Children were excluded if they were born outside Bradford Royal Infirmary (BRI) (as detailed information on birth type and antibiotic administration was unavailable from other sites); withdrew consent or died prior to outcome assessment. Due to small sample sizes in other ethnic categories, the analysis was restricted to children of White British or Pakistani heritage.

### 2.4 Exposure

In the BiB cohort (2007–2011), timing of antibiotic administration was ascertained through manual review of maternity records using a standardised protocol. The medication sequence recorded on anaesthetic charts was prioritised over potentially inaccurate recorded times, and ambiguous cases were reviewed by clinical experts [18]. In the BiBBS cohort (2016–2019), exposure was defined based on the established policy and supported by an audit of maternity records, acknowledging that some misclassification may remain. Uptake of pre‐incision antibiotic administration over calendar time is shown in Figure S1.

### 2.5 Outcomes

Outcome data were obtained from the National Child Measurement Programme (NCMP), which measures the height and weight of primary school children at ages 4–5 years using standardised procedures [19]. BMI was converted to age‐ and sex‐standardised z‐scores using UK90 reference data [20]. Obesity was defined as BMI z‐score > 95th percentile, and BMI z‐score was analysed as a continuous secondary outcome.

### 2.6 Confounder Identification and Adjustment

To guide the selection of confounders for statistical adjustment, a Directed Acyclic Graph (DAG) was constructed, informed by existing literature and clinical expertise (Figure 1). Adjusted models included: maternal characteristics (age at delivery, ethnicity, smoking during pregnancy, education, early‐pregnancy BMI, gestational diabetes, and the number of children aged under 16 years in the household); child characteristics (sex, age‐ and sex‐adjusted birthweight, breastfeeding status, age at BMI measurement at 4–5 years); and antibiotic exposure (maternal prescriptions per trimester and child prescriptions within the first 2 years). Breastfeeding and early‐life antibiotic use were included because they are strong predictors of childhood obesity and vary over calendar time; their inclusion helped address residual confounding not fully captured by adjustment for time. Parity data was not available for the BiBBS cohort, so the number of children in the household was used as a proxy. All models were adjusted a priori for the month and year of delivery as a continuous variable to parsimoniously capture linear secular trends, acknowledging the assumption of linearity across the study period. Data were sourced from study questionnaires and linked maternity and primary care records. Self‐reported ethnicity was sourced from the baseline questionnaire and supplemented with data from primary care records if missing. Early pregnancy BMI was extracted from the maternity information system if recorded within the first 13 weeks of gestation; measurements from ≥ 13 weeks gestation were set to missing, as this is when gestational weight gain typically begins [21].

**FIGURE 1:**
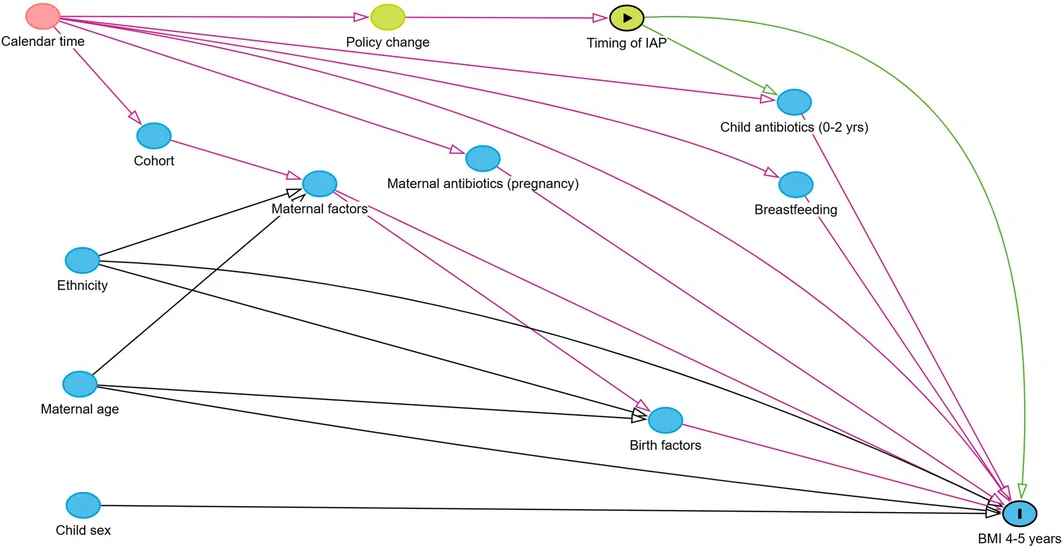
Directed Acyclic Graph (DAG) illustrating the assumed causal structure between intrapartum antibiotic prophylaxis (IAP) timing and childhood obesity at 4–5 years. The DAG includes calendar time, policy change, and cohort membership to reflect the quasi‐experimental design and potential sources of confounding and selection. Breastfeeding and early‐life antibiotic use were included in the adjustment set to account for their strong known associations with childhood obesity, despite their potential role as mediators.

### 2.7 Causal Framework and Assumptions

This study uses a policy‐driven change in IAP timing as a quasi‐experimental exposure. This reduces confounding by indication but is not a formal instrumental variable or interrupted time series design. Causal interpretation therefore assumes that, after accounting for measured covariates and calendar time, there are no unmeasured factors that influence both exposure and childhood BMI, and that no changes over time independently affect the outcome. Breastfeeding and early‐life antibiotic exposure occur after the exposure and may lie on causal pathways. Estimates should be interpreted as conditional on these factors rather than as total causal effects.

### 2.8 Statistical Analysis

Analyses were conducted in Stata (Version 17, StataCorp LLC). Descriptive statistics are presented as mean (SD) or frequency (%). Given the distinct socioeconomic and physiological risk profiles of the White British and Pakistani populations in Bradford established in previous literature [22, 23, 24, 25], we stratified analysis a priori by ethnicity. Multivariable Poisson regression with standard robust errors was used to estimate adjusted risk ratios for obesity, and linear regression for BMI z‐score (adjusted mean difference). Standard errors were clustered by mother. As a sensitivity analysis, models including an interaction term between IAP timing and calendar time were fitted.

### 2.9 Missing Data

Missing covariate data were handled using multiple imputation by chained equations (50 imputed datasets). This method was selected to minimise bias and maximise statistical power, as an initial analysis showed that the data were not missing completely at random (Table S1), indicating that complete case analysis could be biased. Distributions in imputed datasets were consistent with observed data (Table S2).

### 2.10 Ethics Approval

This study received Health Research Authority (HRA) and Health and Care Research Wales (HCRW) approval (ref: 238908). The Born in Bradford project has ethical approval from the Bradford Research Ethics committee (ref: 07/H1302/112). Women recruited to BiB and BiBBS gave informed consent. Consent for their participation and on behalf of their children.

## 3 Results

### 3.1 Participant Characteristics

The final study cohort included 1985 children delivered by CS (Figure 2). Of these, 324 (16.3%) were exposed to pre‐incision antibiotics (the exposed group) and 1661 (83.7%) to post‐cord clamping antibiotics (the unexposed group). Participant characteristics are summarised in Tables 1, S3 details the distribution of participants by cohort of origin and exposure status. The unexposed group was comprised entirely of children from the earlier BiB cohort, while the exposed group included children from the later BiB years (*n* = 61). Temporal patterns in exposure by cohort are shown in Figure S1 and Table S4 provides a stratified comparison of participant characteristics by ethnicity and timing. The overall prevalence of obesity at age 4–5 was 11.9%. Unadjusted prevalence was higher in the exposed group (15.7%) than the unexposed group (11.2%) (Table 1), consistent across sex and ethnic groups (Table S5). In contrast, mean BMI z‐scores were similar between exposure groups, with no consistent differences across subgroups.

**FIGURE 2:**
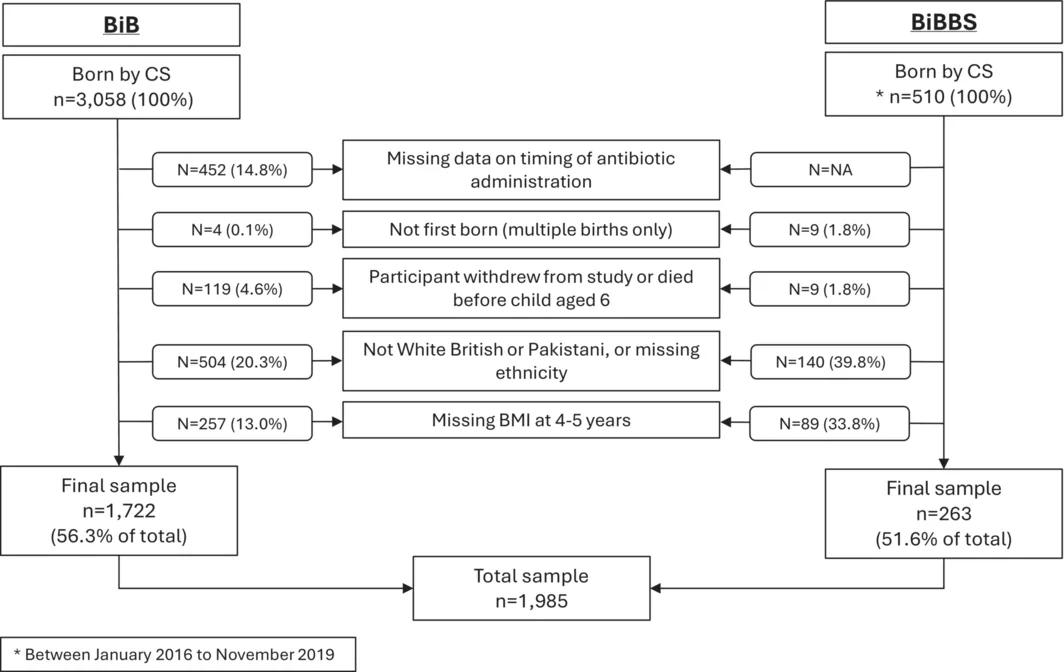
Sample flow chart. Percentages reflect the proportional impact of each eligibility step. Missing data on timing of intrapartum antibiotic prophylaxis occurred only within the Born in Bradford (BiB) cohort, where exposure was ascertained from maternity records during the policy transition period. All children in the Born in Bradford's Better Start (BiBBS) cohort were classified as exposed based on the established hospital policy and therefore could not have missing exposure data. Missing BMI outcome data occurred in both cohorts.

**TABLE 1:** Cohort characteristics overall and by exposure to in utero prophylactic antibiotics. Values are frequency (%) or mean (SD).

	All	Post cord‐clamping	Pre‐incision
	*N* = 1985 (100.0%)	*N* = 1661 (83.7%)	*N* = 324 (16.3%)
Mother characteristics			
Age at delivery	29.2 (SD 5.7)	29.0 (SD 5.7)	30.4 (SD 5.5)
Ethnicity			
White British	863 (43.5%)	798 (48.0%)	65 (20.1%)
Pakistani	1122 (56.5%)	863 (52.0%)	259 (79.9%)
Smoked during pregnancy	252 (14.8%)	220 (15.8%)	32 (10.2%)
Missing	280	269	11
Number of children < 16 at home			
None	854 (44.4)	770 (46.4)	84 (32.1)
1	497 (25.9)	426 (25.6)	71 (27.1)
2	335 (17.4)	274 (16.5)	61 (23.3)
3+	237 (12.3)	191 (11.5)	46 (17.6)
Missing	62	0	62
Educational attainment			
Less than degree level	1214 (72.2%)	1022 (73.4%)	192 (66.2%)
Degree level	468 (27.8%)	370 (26.6%)	98 (33.8%)
Missing	303	269	34
BMI (kg/m^2^)	28.2 (6.6)	28.1 (6.5)	28.5 (7.0)
Missing	556	498	58
Gestational diabetes	193 (12.1)	176 (12.2)	17 (11.6)
Missing	392	214	178
Antibiotics prescribed in first trimester	323 (16.3%)	271 (16.3%)	52 (16.1%)
Antibiotics prescribed in second trimester	335 (16.9%)	278 (16.7%)	57 (17.6%)
Antibiotics prescribed in third trimester	250 (12.6%)	209 (12.6%)	41 (12.7%)
Child characteristics			
Sex			
Male	1070 (53.9%)	899 (54.1%)	171 (52.8%)
Female	915 (46.1%)	762 (45.9%)	153 (47.2%)
Birthweight (Kg)	3.27 (0.56)	3.27 (0.57)	3.29 (0.54)
Birthweight (z‐score)	−0.476 (1.208)	−0.485 (1.214)	−0.429 (1.176)
Missing	63	48	15
Ever breastfed	954 (49.2)	739 (45.2)	215 (70.5)
Missing	45	26	19
Antibiotics prescribed before 2 years of age	1395 (70.3%)	1202 (72.4%)	193 (59.6%)
Age at BMI measurement (mths)	57.6 (SD 4.3)	57.6 (SD 4.4)	58.0 (SD 4.2)
BMI at age 4–5 years (Kg/m^2^)	16.31 (2.10)	16.30 (1.96)	16.38 (2.72)
BMI at age 4–5 years (z‐score)	0.370 (1.142)	0.374 (1.100)	0.346 (1.339)
BMI category at age 4–5 years			
Underweight (< 2nd centile)	31 (1.6)	23 (1.4)	8 (2.5)
Healthy weight (2nd–85th centile)	1461 (73.6)	1227 (73.9)	234 (72.2)
Overweight (> 85th–95th centile)	256 (12.9)	225 (13.6)	31 (9.6)
Obese (> 95th centile)	237 (11.9)	186 (11.2)	51 (15.7)

### 3.2 Association Between Antibiotic Timing and Childhood Body Composition

As shown in Figure 3A, adjusted risk ratios for obesity were 1.25 (95% CI 0.53, 2.98) for White British children and 1.25 (95% CI 0.64, 2.43) for Pakistani children. Adjusted mean differences in BMI z‐score (Figure 3B) were 0.07 (95% CI −0.23, 0.36) and −0.17 (95% CI −0.57, 0.24) respectively. Confidence intervals were wide, indicating limited precision, and the inclusion of an interaction between IAP timing and calendar time did not materially change the estimates.

**FIGURE 3:**
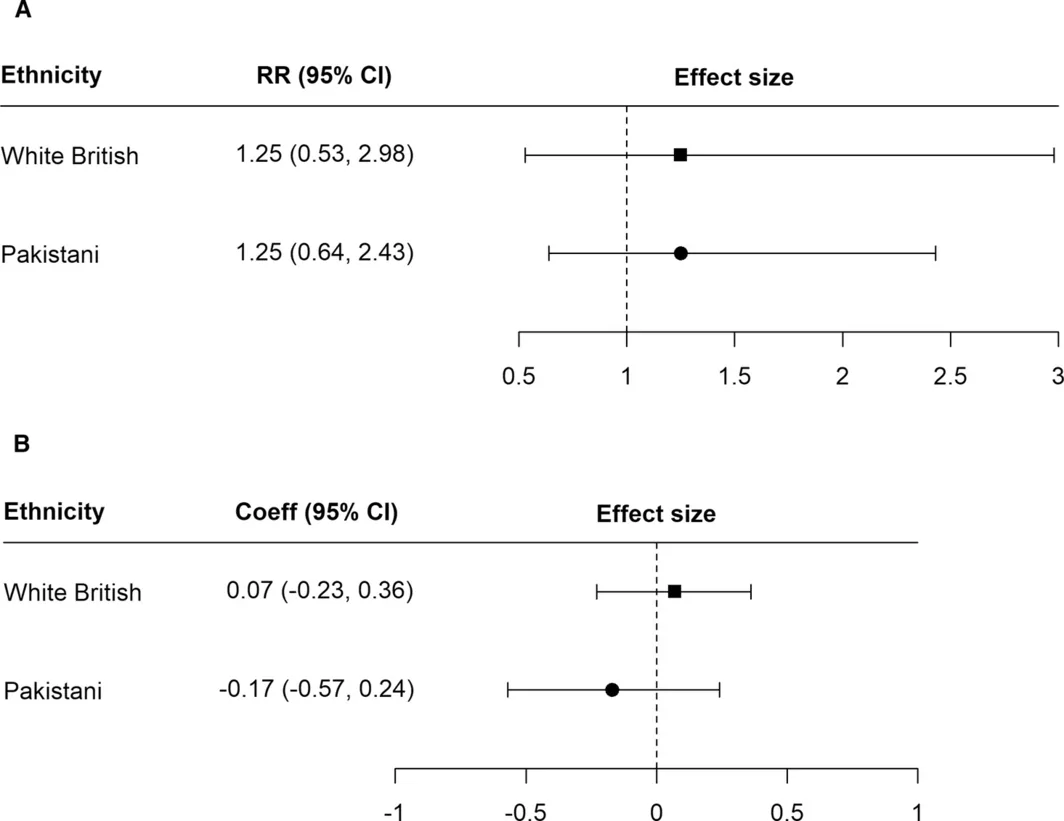
Forest plots of the association between pre‐incision antibiotic prophylaxis and childhood adiposity. The reference group for post‐cord clamping IAP is 1.00 in (A) Obesity. The reference group for (B) BMI Z‐score is 0.00.

### 3.3 Missing Data Analysis

Comparison of participants with complete versus incomplete data indicated the data were not missing completely at random (Table S1). Patterns of missingness differed by ethnicity and maternal characteristics. Among White British women, those excluded were more likely to have no other children in the household. Among Pakistani women, those excluded had a higher BMI, were less likely to breastfeed, and were more likely to have gestational diabetes and higher birthweight infants. These differences suggest that complete case analysis would have been biased; multiple imputation was therefore used.

## 4 Comment

### 4.1 Principal Findings

In this quasi‐experimental study, we did not observe a clear difference in obesity risk or BMI z‐score at 4–5 years between exposure groups. However, confidence intervals were wide, so modest clinically relevant effects cannot be excluded. The divergence between the positive risk ratio for obesity and the negative mean difference in BMI z‐score in the Pakistani group suggests that while the mean BMI shifted slightly downwards, there may be a specific effect at the upper tail of the distribution, or this may reflect uncertainty due to sampling variability.

### 4.2 Strengths of the Study

The key strength of this study is its quasi‐experimental design based on hospital policy change, which was intended to minimise confounding by indication often seen in observational studies of antibiotic use. We utilised data from two large, prospective birth cohorts with linked health records and standardised outcome assessments via the NCMP. Our robust statistical analysis approach, using multiple imputation to handle missing data and stratifying by ethnicity to account for effect modification, allowed us to produce reliable and nuanced results.

### 4.3 Limitations of the Data

Despite these strengths, the study has limitations. The primary limitation relates to the quasi‐experimental nature of the design and the potential for selection bias arising from the use of two distinct cohorts to form the exposure groups. The unexposed group was drawn entirely from the BiB cohort (recruited 2007–2011), while most of the exposed group originated from BiBBS (recruited 2016–2019), which sampled from more socioeconomically deprived areas. As a result, the timing of intrapartum antibiotic prophylaxis is closely aligned with calendar time and cohort membership. Although we adjusted for maternal factors and month/year of birth to account for socioeconomic and secular trends, causal interpretation relies on the assumption that these adjustments adequately capture differences between cohorts and temporal changes; residual confounding due to unmeasured structural or secular factors cannot be excluded. Additionally, data on the type of caesarean section (elective vs. emergency) were unavailable for the BiBBS cohort and could not be included in our models. This is a potential unmeasured confounder, as emergency sections are associated with different maternal and foetal stress profiles compared to elective procedures. We were also unable to adjust for paternal history of obesity, which may represent an additional unmeasured confounder. Our analysis of confounding antibiotic exposures was limited to binary variables, precluding any dose–response analysis. Breastfeeding and early‐life antibiotic exposure were included in adjusted models to account for secular trends and cohort differences not fully captured by calendar time alone. However, as these variables occur after the exposure and may lie on the causal pathways, estimates should be interpreted as conditional on these factors rather than total causal effects. Residual confounding from unmeasured secular trends in diet or lifestyle that occurred in the decade separating the two cohorts cannot be fully excluded, although adjustment for month and year of birth was intended to mitigate this. This may be particularly relevant given the COVID‐19 pandemic, which may have influenced follow‐up and introduced selection bias, as the proportion of children with missing outcome data was higher in the exposed cohort. Due to small sample sizes in the exposed group, the resulting estimates are imprecise, and we cannot exclude the possibility of modest clinically relevant effects.

### 4.4 Interpretation

Our findings provide a counterbalance to previous studies that have reported positive associations between intrapartum antibiotic exposure and childhood BMI. Studies by Koebnick et al. and Sidell et al. found that IAP for Group B Streptococcus was linked to a sustained increase in childhood BMI up to the age of 10 [11, 12, 13]. There are several reasons why our results may differ. Firstly, those studies primarily compared IAP exposure to no antibiotic exposure (in vaginal births) or different types of antibiotics. Our study asks a more specific question: among children all born by CS, does the *timing* of the same prophylactic antibiotic have long‐term consequences? Our findings suggest the critical exposure may be the CS delivery itself, with any additional impact from a single antibiotic dose being minimal or transient.

Importantly, this study is best understood as a quasi‐experimental comparison arising from policy change, rather than a formal natural experiment. While this design reduces confounding by indication—since all women received antibiotics for the same clinical indication (CS) – it does not eliminate the potential for residual confounding, particularly given the close alignment between exposure, calendar time, and cohort membership. As such, the findings should be interpreted as estimates from a policy‐driven comparison that reduce, but do not remove, the risk of bias. Reviews have flagged that evidence linking antibiotics and obesity is of “very low certainty” due to a risk of confounding by indication [9, 10]. By comparing two groups of women receiving antibiotics for the same indication, with variation in timing largely determined by hospital policy, our study reduces this source of bias relative to conventional observational comparisons, although it cannot fully exclude alternative explanations related to secular or cohort differences.

## 5 Conclusion

Our findings are compatible with no large adverse effect of prophylactic antibiotics at caesarean section on the risk of childhood obesity at 4–5 years. Confidence intervals were wide, and modest clinically relevant effects cannot be excluded. These findings should be interpreted in the context of a quasi‐experimental, policy‐driven comparison, which reduces but does not eliminate the potential for residual confounding. Given the well‐established benefit of administering antibiotics prior to incision in reducing maternal surgical site infections, our results are compatible with no large adverse effect on long‐term metabolic outcomes and may provide some reassurance regarding the safety of current clinical guidelines. Future research using larger population‐based datasets is warranted to improve precision and help exclude more modest effects.

## Author Contributions

Gillian Santorelli conducted the statistical analyses and drafted the initial manuscript. Lucy Pembrey, Emily S. Petherick, Sam Oddie, Neil Pearce and John Wright obtained the funding for the study. Sergio Souza da Cunha contributed to the early stages of data preparation. All authors contributed to the interpretation of the findings, critically reviewed the manuscript, and approved the final version.

## Funding

Born in Bradford is supported by a Wellcome programme grant [223601; Born in Bradford: Age of Wonder] and the National Institute for Health Research under its Applied Research Collaboration Yorkshire and Humber, Grant number: NIHR200166. This study was funded by the UK National Institute for Health and Care Research (NIHR) Health Technology Assessment Programme (16/150/06). The views expressed are those of the authors and not necessarily those of the NIHR or the Department of Health and Social Care.

## Consent

Mothers provided informed consent to the linkage of their and their children's clinical records.

## Conflicts of Interest

The authors declare no conflicts of interest.

## Supporting information


**Figure S1:** Proportion of caesarean births receiving pre‐incision antibiotic prophylaxis by year of birth. The figure illustrates uptake of the revised hospital policy over calendar time, including the transition within the BiB cohort and high adherence in later years represented by the BiBBS cohort, where exposure was defined based on the established policy and supported by audit of maternity records.
**Table S1:** Comparison of participant characteristics for those included versus excluded from the complete case analysis.
**Table S2:** Distribution of covariables in the original dataset compared to the pooled multiple imputation dataset.
**Table S3:** Participant characteristics by cohort and timing of intrapartum antibiotic prophylaxis (IAP) at caesarean section.
**Table S4:** Cohort characteristics stratified by ethnicity and timing of intrapartum antibiotic prophylaxis.
**Table S5:** Obesity prevalence and mean BMI z‐score at age 4–5 years, stratified by sex and timing of intrapartum antibiotic prophylaxis.

## Data Availability

The Born in Bradford (BiB) and Born in Bradford's Better Start (BiBBS) data are available to researchers via a managed open access system. Researchers should first consult the Born in Bradford Data Dictionary and Guidance for Collaborators. Enquiries can be directed to borninbradford@bthft.nhs.uk.
