# Supplementary material for: The Effect of Intrapartum Antibiotic Prophylaxis at Caesarean Section on Childhood Obesity: A Quasi‐Experimental Study in Two Bi‐Ethnic UK Cohorts
Source: Paediatr Perinat Epidemiol. 2026 Aug 4;40(8):909–17. doi: 10.1111/ppe.70179 (PMC13763148; doi:10.1111/ppe.70179)
Supplement: Supplementary file 1 [file PPE-40-909-s001.docx]

Figure S1. Proportion of caesarean births receiving pre-incision antibiotic prophylaxis by year of birth. The figure illustrates uptake of the revised hospital policy over calendar time, including the transition within the BiB cohort and high adherence in later years represented by the BiBBS cohort, where exposure was defined based on the established policy and supported by audit of maternity records.

Table S1: Comparison of participant characteristics for those included versus excluded from the complete case analysis.

|  | **All** | | **White British** | | **Pakistani** | |
| --- | --- | --- | --- | --- | --- | --- |
| **Covariables** | **Included**  **n=1,003 (50.5%)** | **Excluded**  **n=982 (49.5%)** | **Included**  **n=506 (58.6%)** | **Excluded**  **n=357 (41.4%)** | **Included**  **n=497 (44.3%)** | **Excluded**  **n=625 (55.7%)** |
| **Maternal characteristics** |  |  |  |  |  |  |
| Age at delivery | 29.2 (SD 5.7) | 29.3 (SD 5.8) | 29.1 (SD 5.9) | 28.8 (SD 6.3) | 29.3 (SD 5.4) | 29.5 (SD 5.5) |
| Smoking during pregnancy | 15.0% | 14.4% | 26.5% | 32.5% | 3.4% | 4.6% |
| Number of children |  |  |  |  |  |  |
| None | 38.6% | 50.8% | 48.0% | 63.3% | 29.0% | 43.1% |
| 1 | 29.4% | 22.0% | 34.0% | 22.5% | 24.8% | 21.6% |
| 2 | 18.7% | 16.0% | 13.0% | 10.0% | 24.6% | 19.7% |
| 3 | 13.3% | 11.3% | 4.9% | 4.3% | 21.7% | 15.6% |
| Education – degree level | 27.6% | 28.1% | 23.3% | 23.8% | 32.0% | 30.6% |
| BMI (kg/m^2^) | 28.0 (SD 6.5) | 28.5 (SD 6.7) | 28.8 (SD 6.6) | 28.1 (SD 7.1) | 27.2 (SD 6.3) | 28.7 (SD 6.5) |
| Gestational diabetes | 12.6% | 11.4% | 7.3% | 4.6% | 19.5% | 15.0% |
| Antibiotics prescribed in first trimester | 17.9% | 14.7% | 16.2% | 14.0% | 16.7% | 19.2% |
| Antibiotics prescribed in second trimester | 16.1% | 17.7% | 15.4% | 15.1% | 12.7% | 13.6% |
| Antibiotics prescribed in third trimester | 12.8% | 12.4% | 12.9% | 10.4% | 12.8% | 12.4% |
| **Child characteristics** |  |  |  |  |  |  |
| Male | 52.9% | 54.9% | 52.8% | 57.1% | 53.1% | 53.1% |
| Birthweight (Kg) | 3.27 (SD 0.56) | 3.27 (SD 0.56) | 3.41 (SD 0.56) | 3.37 (SD 0.56) | 3.14 (SD 0.54) | 3.21 (SD 0.55) |
| Birthweight (z-score) | -0.47 (SD 1.21) | -0.49 (SD 1.20) | -0.18 (SD 1.19) | -0.28 (SD 1.20) | -0.76 (SD 1.17) | -0.60 (SD 1.19) |
| Every breastfed | 50.2% | 48.0% | 42.3% | 40.5% | 58.4% | 52.3% |
| Antibiotics prescribed before 2 years of age | 71.4% | 69.1% | 69.8% | 65.6% | 73.0% | 71.2% |
| BMI at age 4-5 years (Kg/m^2^) | 16.35 (SE 0.06) | 16.27 (SE 0.07) | 16.56 (SD 1.79) | 16.36 (SD 1.72) | 16.12 (SD 2.06) | 16.22 (SD 2.50) |
| BMI at age 4-5 years (z-score) | 0.41 (SD 1.09) | 0.33 (SD 1.19) | 0.57 (SD 0.94) | 0.44 (SD 0.99) | 0.24 (SD 1.20) | 0.26 (SD 1.29) |
| BMI category at age 4-5 years |  |  |  |  |  |  |
| Underweight (<2^nd^ centile) | 1.3% | 1.8% | 0.4% | 0.8% | 2.2% | 2.4% |
| Healthy weight (2^nd^-85^th^ centile) | 73.7% | 73.5% | 72.3% | 72.8% | 75.1% | 73.9% |
| Overweight (>85^th^-95^th^ centile) | 13.6% | 12.2% | 16.4% | 16.5% | 10.7% | 9.8% |
| Obese (>95^th^ centile) | 11.5% | 12.4% | 10.9% | 9.8% | 12.1% | 13.9% |

Results are presented overall and stratified by maternal ethnicity. P-values were derived from chi-squared tests for categorical variables and independent t-tests for continuous variables.

Table S2: Distribution of covariables in the original dataset compared to the pooled multiple imputation dataset.

| **Covariables** | **N missing (%)** | **Observed** | **Imputed** |
| --- | --- | --- | --- |
| Smoking during pregnancy | 280 (14.1%) |  |  |
| No |  | 85.5% | 85.3% |
| Yes |  | 14.5% | 14.7% |
| Number of children in household | 62 (3.1%) |  |  |
| None |  | 43.9% | 44.4% |
| 1 |  | 25.8% | 25.8% |
| 2 |  | 17.6% | 17.4% |
| 3 |  | 12.7% | 12.3% |
| Education | 303 (15.3%) |  |  |
| Less than degree level |  | 70.9% | 72.2% |
| Degree level |  | 29.1% | 27.8% |
| Maternal BMI | 556 (28.0%) | 28.1 (SE 0.2) | 28.2 (SE 0.2) |
| Gestational diabetes | 392 (19.8%) |  |  |
| No |  | 87.5% | 87.9% |
| Yes |  | 12.5% | 12.1% |
| Birthweight (Kg) | 63 (3.2%) | 3.27 (SE 0.01) | 3.27 (SE 0.01) |
| Every breastfed | 45 (2.3%) |  |  |
| No |  | 50.6% | 50.8% |
| Yes |  | 49.4% | 49.2% |

Table S3: Participant characteristics by cohort and timing of intrapartum antibiotic prophylaxis (IAP) at caesarean section.

|  | **BiB cohort** | | **BiBBS cohort** |
| --- | --- | --- | --- |
|  | **Post cord clamping** | **Pre-incision** | **Pre-incision** |
|  | N=1,661  (96.5%) | N=61  (3.5%) | N=263  (100.0%) |
| **Mother characteristics** |  |  |  |
| Age at delivery | 29.0 (SD 5.7) | 29.3 (SD 5.3) | 29.3 (SD 5.3) |
| Ethnicity |  |  |  |
| White British | 798 (48.0%) | 29 (47.5%) | 36 (13.7%) |
| Pakistani | 863 (52.0%) | 32 (52.5%) | 227 (86.3%) |
| Smoked during pregnancy |  |  |  |
| No | 1,172 (84.2%) | 43 (86.0%) | 238 (90.5%) |
| Yes | 220 (15.8%) | 7 (14.0%) | 25 (9.5%) |
| Missing | 269 | 11 | 0 |
| Number of children <16 at home |  |  |  |
| None | 770 (46.4%) | 36 (59.0%) | 48 (23.9%) |
| 1 | 426 (25.7%) | 13 (21.3%) | 58 (28.9%) |
| 2 | 274 (16.5%) | 8 (13.1%) | 53 (26.4%) |
| 3+ | 191 (11.5%) | 4 (6.6%) | 42 (20.9%) |
| Missing | 0 | 0 | 62 |
| Educational attainment |  |  |  |
| Less than degree level or equivalent | 1,022 (73.4%) | 34 (68.0%) | 158 (65.8%) |
| Degree level or equivalent | 370 (26.6%) | 16 (32.0%) | 82 (34.2%) |
| Missing | 269 | 11 | 23 |
| BMI (kg/m^2^) | 28.1 (SD 6.5) | 28.0 (SD 8.5) | 28.6 (SD 6.7) |
| Missing | 498 | 23 | 35 |
| Gestational diabetes | 176 (12.2%) | 4 (7.3%) | 13 (14.3%) |
| Missing | 214 | 6 | 172 |
| Antibiotics prescribed in 1^st^ trimester | 271 (16.3%) | 4 (6.6%) | 48 (18.3%) |
| Antibiotics prescribed in 2^nd^ trimester | 278 (16.7%) | 12 (19.7%) | 45 (17.1%) |
| Antibiotics prescribed in 3^rd^ trimester | 209 (12.6%) | 15 (24.6%) | 26 (9.9%) |
| **Child characteristics** |  |  |  |
| Sex |  |  |  |
| Male | 899 (54.1%) | 33 (54.1%) | 138 (52.5%) |
| Female | 762 (45.9%) | 28 (45.9%) | 125 (47.5%) |
| Birthweight (Kg) | 3.27 (SD 0.57) | 3.36 (SD 0.53) | 3.28 (SD 0.54) |
| Birthweight (SDS) | -0.48 (SD 1.21) | -0.46 (SD 1.18) | -0.46 (1.18) |
| Missing | 48 | 2 | 13 |
| Ever breastfed | 739 (45.2%) | 27 (44.3%) | 188 (77.1%) |
| Missing | 26 | 0 | 19 |
| Antibiotics prescribed before 2 years of age | 1,202 (72.4%) | 43 (70.5%) | 150 (57.0%) |
| Age at BMI measurement (mths) | 57.6 (4.4) | 58.0 (4.2) | 58.0 (4.2) |
| BMI at age 4-5 years (kg/m^2^) | 16.3 (SD 2.0) | 16.1 (SD 2.0) | 16.4 (SD 2.9) |
| BMI at age 4-5 years (SDS) | 0.37 (SD 1.10) | 0.26 (SD 1.25) | 0.37 (SD 1.36) |
| BMI category |  |  |  |
| Underweight (<2^nd^ centile) | 23 (1.4%) | 2 (3.3%) | 6 (2.3%) |
| Healthy weight (2^nd^-85^th^ centile) | 1,227 (73.9%) | 44 (72.1%) | 190 (72.2%) |
| Overweight (>85^th^-95^th^ centile) | 225 (13.6%) | 7 (11.5%) | 24 (9.1%) |
| Obese (>95^th^ centile) | 186 (11.2%) | 8 (13.1%) | 43 (16.4%) |

Values are frequency (%) or mean (SD)

Table S4: Cohort characteristics stratified by ethnicity and timing of intrapartum antibiotic prophylaxis.

|  | **White British** | | **Pakistani** | |
| --- | --- | --- | --- | --- |
|  | **Post cord clamping** | **Pre-incision** | **Post cord clamping** | **Pre-incision** |
|  | N=798 (92.5%) | N=65 (7.5%) | N=863 (76.9%) | N=259 (23.1%) |
| **Mother characteristics** |  |  |  |  |
| Age at delivery | 29.0 (SD 6.1) | 28.8 (SD 6.2) | 29.0 (SD 5.4) | 30.8 (SD 5.3) |
| Smoked during pregnancy |  |  |  |  |
| No | 499 (72.0%) | 39 (66.1%) | 673 (96.3%) | 242 (95.3%) |
| Yes | 194 (28.0%) | 20 (33.9%) | 26 (3.7%) | 12 (4.7%) |
| Missing | 105 | 6 | 164 | 5 |
| Number of children <16 at home |  |  |  |  |
| None | 440 (55.1%) | 25 (42.4%) | 330 (38.2%) | 59 (29.1%) |
| 1 | 234 (29.3%) | 17 (28.8%) | 192 (22.3%) | 54 (26.6%) |
| 2 | 93 (11.7%) | 8 (13.6%) | 181 (21.0%) | 53 (26.1%) |
| 3+ | 31 (3.9%) | 9 (15.3%) | 160 (18.5%) | 37 (18.2%) |
| Missing | 0 | 6 | 0 | 56 |
| Educational attainment |  |  |  |  |
| Less than degree level or equivalent | 526 (75.9%) | 48 (73.8%) | 496 (71.0%) | 144 (61.8%) |
| Degree level or equivalent | 167 (24.1%) | 9 (15.8%) | 203 (29.0%) | 89 (38.2%) |
| Missing | 105 | 8 | 164 | 26 |
| BMI (kg/m^2^) | 28.6 (SD 6.6) | 28.9 (SD 8.0) | 27.5 (SD 6.3) | 28.4 (SD 6.8) |
| Missing | 208 | 17 | 290 | 41 |
| Gestational diabetes | 44 (6.4%) | 3 (7.7%) | 132 (17.4%) | 14 (13.9%) |
| Missing | 111 | 26 | 103 | 152 |
| Antibiotics prescribed in 1^st^ trimester | 123 (15.4%) | 9 (13.9%) | 148 (17.2%) | 43 (16.6%) |
| Antibiotics prescribed in 2^nd^ trimester | 121 (15.2%) | 11 (16.9%) | 157 (18.2%) | 46 (17.8%) |
| Antibiotics prescribed in 3^rd^ trimester | 93 (11.7%) | 9 (13.9%) | 116 (13.4%) | 32 (12.4%) |
| **Child characteristics** |  |  |  |  |
| Sex |  |  |  |  |
| Male | 435 (54.5%) | 36 (55.4%) | 464 (53.8%) | 135 (52.1%) |
| Female | 363 (45.5%) | 29 (44.6%) | 399 (46.2%) | 124 (47.9%) |
| Birthweight (Kg) | 3.39 (SD 0.56) | 3.39 (SD 0.57) | 3.16 (SD 0.55) | 3.27 (SD 0.53) |
| Birthweight (SDS) | -0.22 (SD 1.19) | -0.24 (1.26) | -0.73 (SD 1.18) | -0.48 (1.15) |
| Missing | 20 | 3 | 28 | 12 |
| Ever breastfed | 317 (40.4%) | 34 (56.7%) | 422 (49.6%) | 181 (73.9%) |
| Missing | 14 | 5 | 12 | 14 |
| Antibiotics prescribed before 2 years of age | 550 (68.9%) | 37 (56.9%) | 652 (75.6%) | 156 (60.2%) |
| Age at BMI measurement (mths) | 57.7 (4.2) | 58.6 (3.7) | 57.5 (4.5) | 57.8 (4.3) |
| BMI at age 4-5 years (kg/m^2^) | 16.5 (SD 1.8) | 16.5 (SD 1.8) | 16.1 (SD 2.1) | 16.3 (SD 2.9) |
| BMI at age 4-5 years (z-score) | 0.52 (SD 0.95) | 0.55 (SD 1.05) | 0.24 (SD 1.21) | 0.29 (SD 1.40) |
| BMI category |  |  |  |  |
| Underweight (<2^nd^ centile) | 5 (0.6%) | 0 | 18 (2.1%) | 8 (3.1%) |
| Healthy weight (2^nd^-85^th^ centile) | 580 (72.7%) | 46 (70.8%) | 647 (75.0%) | 188 (72.6%) |
| Overweight (>85^th^-95^th^ centile) | 132 (16.5%) | 10 (15.4%) | 93 (10.8%) | 21 (8.1%) |
| Obese (>95^th^ centile) | 81 (10.2%) | 9 (13.9%) | 105 (12.2%) | 42 (16.2%) |

Values are frequency (%) or mean (SD)

Table S5: Obesity prevalence and mean BMI z-score at age 4-5 years, stratified by sex and timing of intrapartum antibiotic prophylaxis.

|  | **Boys** | | **Girls** | |
| --- | --- | --- | --- | --- |
|  | **Post cord clamping** | **Pre-incision** | **Post cord clamping** | **Pre-incision** |
| Obesity, n (%) | | | | |
| Overall | 92 (10.2%) | 27 (15.8%) | 94 (12.3%) | 24 (15.7%) |
| White British | 37 (8.5%) | 5 (13.9%) | 44 (12.1%) | 4 (13.8%) |
| Pakistani | 55 (11.9%) | 22 (16.3%) | 50 (12.5%) | 20 (16.1%) |
| BMI z-score, mean (SD) | | | | |
| Overall | 0.36 (1.07) | 0.29 (1.31) | 0.39 (1.13) | 0.41 (1.38) |
| White British | 0.48 (0.90) | 0.65 (0.84) | 0.57 (1.01) | 0.42 (1.28) |
| Pakistani | 0.25 (1.20) | 0.19 (1.39) | 0.23 (1.21) | 0.41 (1.40) |
